# Slower Time estimation in Post-Traumatic Stress Disorder

**DOI:** 10.1038/s41598-017-18907-5

**Published:** 2018-01-10

**Authors:** Carmelo M. Vicario, Kim L. Felmingham

**Affiliations:** 1 School of Psychology, University of Tasmania, Hobart, TAS Australia; 2 School of Psychological Sciences, University of Melbourne, Melbourne, Australia

## Abstract

Cognitive deficits in Posttraumatic Stress Disorder (PTSD) and dissociative symptoms suggest there may be an underlying and persistent problem with temporal processing in PTSD, but this question has not been systematically examined. We investigated the ability of a group of PTSD participants in estimating the duration of supra-second visual stimuli relative to healthy controls. **The data of 59** participants with PTSD and **62** healthy controls**, collected from the BRID database, have been examined**. Overall, our results indicate that PTSD patients overestimate the duration of **the displayed** stimuli. Moreover, we found that PTSD are more variable in the time estimation compared to the control group. Finally, we found evidence that working memory and attention impairments were associated with time overestimation in PTSD. The finding of time overestimation in PTSD accords with previous reports of time overestimation during stressful experiences associated with fear and arousal, but extends findings to suggest **it** remains in chronic PTSD populations processing non-emotional stimuli. The evidence of time overestimation in PTSD suggests the potential relevance of this factor as a cognitive marker in assessing the neuropsychological profile of this clinical population.

## Introduction

Posttraumatic Stress Disorder (PTSD) is an anxiety disorder affecting approximately 6.8% of people at some time in their life (e.g., refs^1,2^. See also^3^ for a recent review). PTSD is characterized by intrusive memories, which are typically fragmented memories of the trauma frozen in time, cognitive deficits such as working memory (WM) impairments, information processing speed and attentional biases to threat, and cognitive and behavioural avoidance^4^. Dissociative symptoms can also be prominent in individuals with PTSD, and include a sense of derealisation and depersonalization^5^. This constellation of symptoms suggests there may be a dysregulation in temporal processing or time perception associated with PTSD. Individuals with PTSD often report a slowing of time perception during their trauma^6^, but whether time processing deficits persist in chronic PTSD remains unknown.

Previous research in healthy controls has revealed that an overestimation of time is associated with negative affect and high levels of arousal. In prospective time-estimation studies, viewing negatively-valence images in high arousal states was associated with over-estimated time perception, whereas viewing positive images under high arousal was associated with time underestimation^7^. Similarly, threatening high arousal images were overestimated in duration more than non-threat high arousal stimuli^8^. In retrospective time-estimation studies, Loftus found participants overestimated the duration of a stressful video of a bank robbery^9^. These findings accord with an Approach/Avoidance model of time estimation which suggests there is a slowing of time perception (overestimation of time) in high arousal situations as the outcome is more strenuously anticipated in high arousal/threat situations^7,10^. Campbell & Bryant^10^ tested this model by assessing fear and excitement levels in novice skydivers and their time estimations of the jump on landing. They found a positive association between levels of fear prior to and during the skydive, and estimation of subjective time for the jump, with increased fear associated with an overestimation of time. Finally, there is evidence^11,12^ for a perception of slowed passage of time in individuals with high levels of anxiety.

It is possible that individuals with PTSD may have an underlying disturbance in temporal processing independently of emotional or stressful contexts. There is robust evidence of impairments in WM, executive functioning and attention in PTSD, as confirmed by a recent meta-analysis of 60 studies^13^. Both WM and attention are considered essential for accurate time keeping, as documented by previous investigations in healthy humans and clinical populations (e.g., refs^14–20^).

In further support of the hypothesis of time processing deficits in PTSD is the evidence of structural and functional alterations in a number of brain regions of individuals with PTSD such as the dorsolateral prefrontal cortex (PFC)^21,22^, the superior parietal regions^23,24^, the insula (refs^22,25^. See ref.^26^ for a review) and the basal ganglia^22,25^. All these regions are considered essential in mediating the conscious and unconscious experience of time (refs^27–30^. See refs^31,32^, for reviews). The evidence of dopaminergic alteration in PTSD (e.g., refs^33,34^) adds a neurochemical rationale to the hypothesis of timing deficits in this clinical population, according to the research linking dopamine with the internal clock functions^17,35–39^.

To date, no study has systematically examined the possibility of timing deficits in PTSD in an experimental setting, although a previous report^40^ has included the evaluation of time processing skills in PTSD participants, as a minor part of the study. Furthermore, no studies have examined whether timing deficits are associated with cognitive deficits (such as WM and attentional switching) in PTSD. A psychophysics investigation of time keeping skills in PTSD is timely because it would clarify the suggestion of timing alterations in these patients, documented via clinical interview and demographics questionnaires (e.g., ref.^10^). Moreover, it would expand our current knowledge about the cognitive deficits associated with this clinical disorder.

We explored time processing in PTSD by comparing the ability of a group of these patients and a group of healthy controls in the execution of a supra-second visual timing task. Our analysis included several measures for WM and attentional switching, to investigate any role of these cognitive variables on timing skills. Following from evidence that high arousal and stressful situations are associated with an overestimation of time, or temporal slowing, we expected to detect a timing overestimation in PTSD relative to controls. Further, we expected to find significant relationships between timing performance and both WM/attentional switching, given the role of these cognitive abilities, which are found to be altered in PTSD^13^, in time keeping functions^17,41^.

## Method

### Participants

Data from 61 PTSD and 68 healthy control individuals were extracted from the Brain Resource International Database (BRID, http://www.brainnet.net/about/brain-resource-international-database/ **)**. This database contains data from multiple laboratories (New York, Rhode Island, Nijmegen, London, Adelaide, and Sydney) that have been acquired using standardized data acquisition techniques for cognitive tasks (INTEGNeuro) including the time estimation task (described below). Inter-lab reliability and test-retest reliability measures are high as documented in previous works (e.g., refs^42,43^). Participants were aged between 18 and 65 and matched on gender, age and education. The exclusion criteria of this database included a personal or family history of mental illness, brain injury, neurological disorder, serious medical condition, drug/alcohol addiction, first-degree relative with bipolar disorder, schizophrenia, or genetic disorder. Our study included a sample of 61 patients (32 males, mean age = 42.34 ± 11.60; mean education = 12.92 ± 3.53) and 68 healthy controls (34 males, mean age = 41.75 ± 12.52; mean education = 13.63 ± 3.15). No significant between group difference is reported with regard to age (p = 0.775) and education (p = 0.333) variables. All participants gave written informed consent. The study was approved by the Tasmanian health and Medical Research Ethics committee and at the University of Tasmania (Ref N. H0016534). All methods were performed in accordance with the relevant guidelines and regulations from our Institution and the Tasmanian health and Medical Research Ethics committee.

### Psychometric measures

PTSD diagnoses and measurement of disorder severity were made using the Clinician Administered PTSD scale (CAPS)^44^. WM, speed of information processing and attentional switching performance have been examined by using the Digit span and a computerized version of the Trails Making Test Part A and B as part of the Integneuro battery. The computerized testing protocol of Ïntegneuro has established reliability and validity statistics^42^. IntegNeuro has been developed in accordance with American Psychiatric Association guidelines, it has been validated against traditional pencil and paper tests (and has established test-retest reliability amongst adults^42^. Overall, test-retest reliability is 0.75 (Integ Neuro: Assessment Manual 1.0, Brain Resource Ltd, 2009). Standardization norms have been established in over 1000 healthy participants and these norms form part of the Brain Resource International Database^45^. The cognitive tests were administered using pre-recorded task instructions (via headphones), and responses were given via a touch screen computer or.wav files for spoken answers.

### Tasks

Participants were seated in a sound attenuated room in front of a touchscreen computer (NEC MultiSync LCD 1530 V). All participants completed the cognitive tests as part of a reliable and valid computerized test battery^42,43^. Tests were administered using prerecorded task instructions (via headphones) and computerized and voice recording was used for answers. All participants performed a practice trial before the formal completion of the proposed tasks.

### Time estimation task

A black circle appears on the screen, turning green for times varying between 1 and 12 seconds, in steps of 1 second, in pseduo-random order and for a total of 12 intervals. Each participant was required to attend the screen and estimate the duration of the target trace on the screen, using keys on a fixed display touchpad at the bottom of the screen with the duration range between 1–12 seconds. Each temporal switch was presented once. Therefore, the number of trials was 12.

### Digit Span task

Participants are presented with a series of digits on the touchscreen, separated by a one-second interval. The subject is then immediately asked to enter the digits on a numeric keypad on the touch-screen. In the first part of the test, subjects are required to recall the digits in forward order and reverse order in the second. In each part, the number of digits in each sequence is gradually increased from 3 to 9, with two sequences at each level. The dependent measure is the total number of correct trials forward and backward. The maximum task duration was approximately 6 minutes, with a total of 14 trials. More details about this task are described in a previous report^46^.

### Switching of Attention task

This modified version of the Trail Making Test consisted of two parts. The first, a measure of psychomotor speed, required the connecting of numbers in ascending sequence (i.e., 1–2–3, etc.) (Switching of Attention—Number). The second, a measure of speeded cognitive flexibility, required participants to connect numbers and letters in an ascending but alternating sequence (i.e., 1-A-2-B). Time for completion for each part served as dependent variables. Task duration was approximately 3 minutes with a total of 25 trails. More details about this task are provided in a previous report^46^.

### Data Analysis

Participants’ task performance was evaluated by considering the *proportional bias* (PB) score, which provides a measure of the estimation accuracy calculated from the twelve temporal intervals, where the bias for each trial is calculated as a positive or negative percentage of the actual presented interval; PB is estimated from the absolute value of the average difference between the actual duration of the stimulus and the participant-estimated duration. Thus, an overall positive score (i.e., >0) indicates a time overestimation; while an overall negative score (i.e., <0) indicates a temporal underestimation. We also calculated the *estimation bias variability* (EBV) score, which represents the standard deviation average of the proportional bias. PB and EBV scores of our clinical and control samples were compared by using a two-tailed t-test comparison. Two further analyses we calculated to evaluate in more detail (i.e., for each temporal interval) the timing performance: The raw *time estimation* (TE) mean associated for the twelve temporal intervals (this measure shows, trial by trial, the participants ‘trend in estimating the duration of the presented stimuli).

The *coefficient of variation* (CV), which provides an index of the samples variability in estimating the presented stimuli, was calculated as the ratio between the SD and mean values of TE for each condition^47^. TE and CV were entered into separate groups (PTSD, controls) of 12 data points (1, 2, 3, 4, 5, 6, 7, 8, 9, 10, 11, 12 seconds – temporal intervals) repeated measures Analyses of Variance (ANOVA). Between group differences on the sub-tests adopted to evaluate WM and attention were evaluated by using two tailed t-test. Details about this analysis are reported in Table 1. Finally, Pearson correlation analyses were implemented to measure any relationship between timing performance and cognitive measures of our participants. These further analyses were performed using PB and EBV scores. All Post-hoc comparisons were performed via t-test Bonferroni corrected, and, for all statistical analyses, a p value of <0.05 was considered to be significant. Participants with outlier trials (i.e., ≥3.5 SD from the average) were removed from the analysis. According to this criterion the final ANOVA was conducted on 59 PTSD and 62 controls. Data analysis was performed using Statistica software, version 8.0, Stat Soft, Inc., Tulsa, USA.

**Table 1:** The table reports the mean scores of the examined cognitive variables in PTSD and control participants and the corresponding between groups difference according to the t-test analysis.

**Measures**	**Number of examined control participants**	**Number of examined PTSD participants**	**Mean Controls**	**Mean PTSD**	**t-value**	**Df**	**p-level**
Digitot	62	59	7.47	6.42	2.181	119	*p* = 0.031*
Digitsp	62	59	6.26	5.80	1.548	119	*p* = 0.124
Rdigitot	61	58	4.66	3.45	2.628	117	*p* = 0.009*
Rdigitsp	61	58	4.75	4.05	2.279	117	*p* = 0.024*
Swat_D	62	59	21844,97	26278,61	−2.515	119	*p* = 0.013*
Swae_D	62	59	1.00	1.29	−0.588	119	*p* = 0.557
Swat_DL	61	58	45565,35	57907,99	−3.785	117	*p* < 0.001*
Swae_DL	61	58	1.20	1.62	−1.057	117	*p* = 0.292
SaRt	62	59	493.06	595.64	−4.398	119	*p* < 0.001*
SaRtSd	62	59	121.95	158.42	−2.937	119	*p* = 0.003*
SaFa	62	59	0.47	1.34	−2.157	119	*p* = 0.032*
SaFm	62	59	1.00	2.61	−3.143	119	*p* = 0.002*
Satot	62	59	1.47	3.95	−2.953	119	*p* = 0.003*

*Indicates a significant result. *Acronyms legend*: Digitot (digit span forward, correct trials); Digitsp (digit span forward, recall span); Rdigitot (digit span reverse, correct trials); Rdigitsp (digit span reverse, recall span); Swat_D (switching of attention, completion time - digits); Swae_D (switching of attention, errors - digits); Swat_DL (switching of attention, completion time - digits + letters) Swae_DL (switching of attention, errors - digits + letters); SaRt (Sustained attention Reaction time); SaRtSd (Sustained attention Reaction time variability); SaFa (Sustained attention, false alarm); SaFm (Sustained attention, false misses); Satot (Sustained attention, total errors).

### Data Availability

The data are deposited at the Brain Resource International Database located in Sydney, Australia (BRID, http://www.brainnet.net/about/governance-and-management/). Data can be obtained by contacting the BRAINnet Foundation administrator at michelle.wang@brainnet.net.

## Results

Table 1 provides details on the performance means associated with the cognitive tests between the groups and the between groups statistical comparisons. Although we included 59 PTSD and 62 controls in regard to the timing task paragraph, the performance scores on some WM and attention subtests of 2 participants (1 PTSD and 1 control) were missing. Details about the number of participants evaluated for each single test are provided in Table 1.

The t-test analysis reveals a significant between group difference for the PB score which was negative for the control group (*M* = −0.11) compared to PTSD (*M* = 0.00), *t*_119_ = −3.264, *p* = 0.001. Moreover, we found a higher PBV for the PTSD group (*M* = 0.21) compared to controls (*M* = 0.12), *t*_119_ = 3.534, *p* < 0.001.

### Time Estimation (TE)

**Figure 1:**
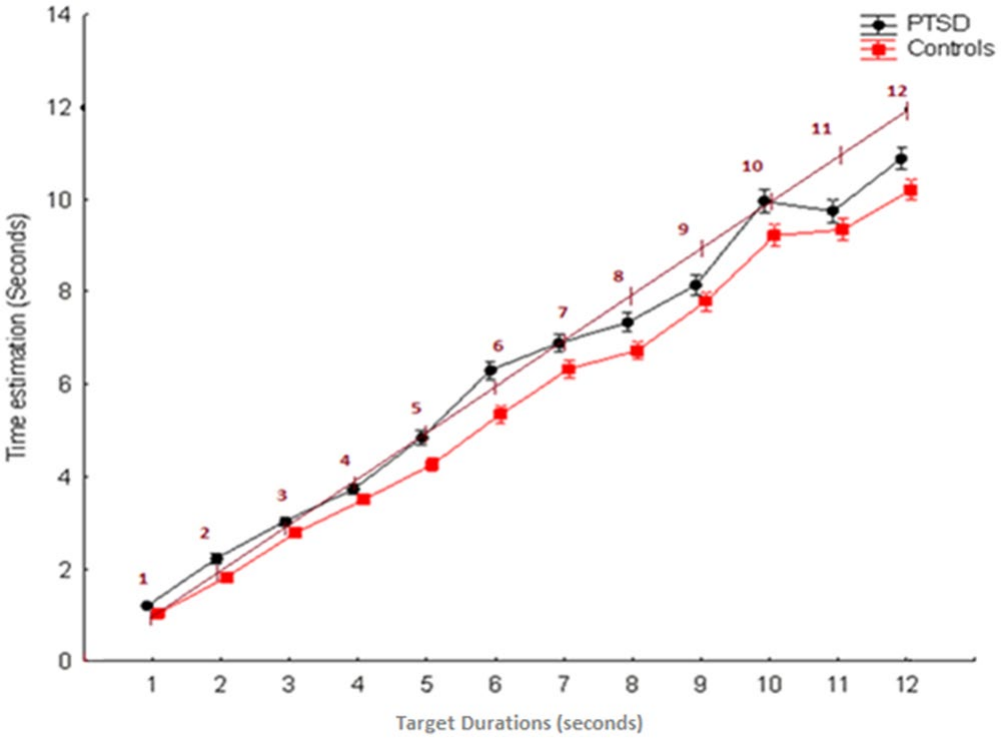
The figure plots temporal estimations associated to the PTSD and control groups for the twelve temporal intervals. The inclined line allows you to observe the shift between the real durations the presented stimuli and the estimations provided by patients and control participants.

The ANOVA detected a significant main effect of the *Group F*_1,118_ = 6.78, *p* = 0.010, *ηp²* = 0.054, *Observed Power* = 0.733, which documents a higher average temporal estimation in the PTSD group (*M* = 6.186 ± 0.135), compared to controls (*M* = 5.694 ± 0.131). The *Temporal Interval factor* F_11,1298_ = 1326.8, *p* < 0.001, *ηp²* = 0.918, *Observed Power* = 1.000 was also significant, as expected. There was also a marginally significant group x temporal interval *F*_11,1298_ = 1.778, *p* = 0.053*, ηp²* = 0.014, *Observed Power* = 0.865 interaction term. See Fig. 1 for details.

### Coefficient of Variation (CV)

**Figure 2:**
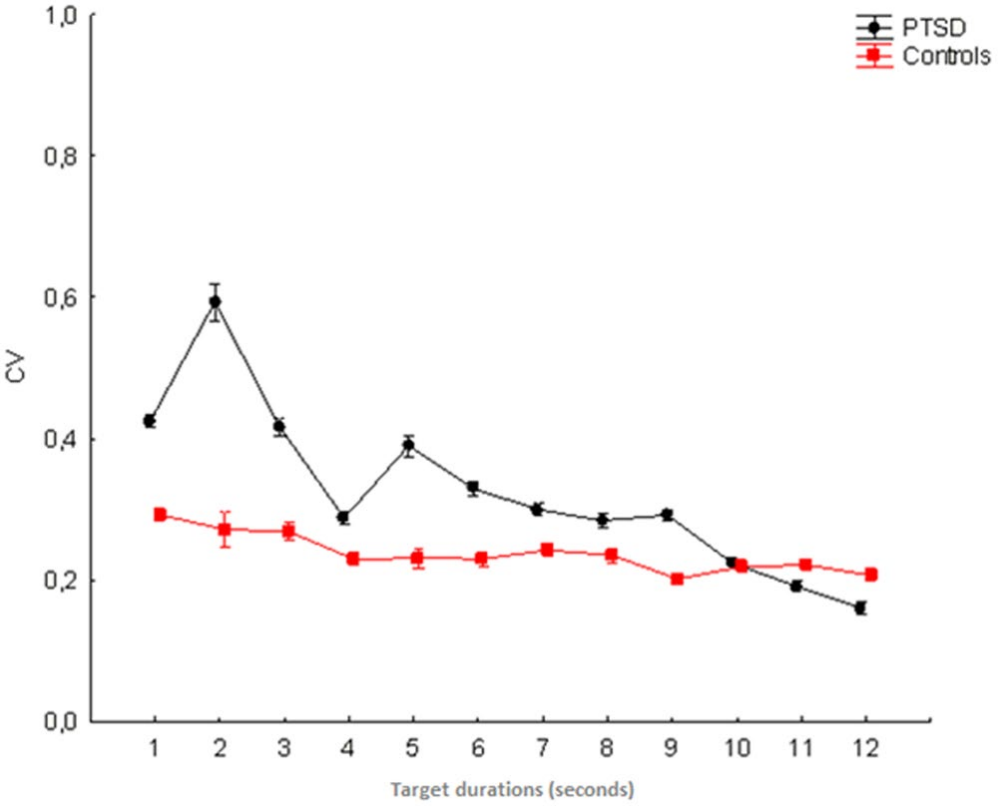
The figure plots data on coefficient of variation (CV) scores associated to PTSD and control participants.

The ANOVA detected a significant main effect of the *Group F*_1,118_ = 53.80, *p* < 0.001, *ηp²* = 0.313, *Observed Power* = 1.000, which documents higher variability in the temporal estimation of the PTSD group (*M* = 0.324 ± 0.008), compared to controls (*M* = 0.237 ± 0.008). The *Temporal Interval* factor *F*_11,1298_ = 112.47, *p* < 0.001, *ηp²* = 0.509, *Observed Power* = 1.000 was also significant. Finally, we documented a significant group × temporal interval *F*_11,1298_ = 60.58, *p* < 0.001, *ηp²* = 0.339, *Observed Power* = 1.000 interaction term. Although Fig. 2 shows higher PTSD variability through the 1–9 seconds range, the post-hoc comparison documents significant between groups differences (*p* < 0.001) only for 1, 2, 3, 5, 6, 9 temporal intervals. See Fig. 2 for details.

### Correlations

#### PTSD

Results document negative correlations between PB and all the digit span measures (*p* ≥ 0.012 and ≤0.028, see Table 2 for details). This suggests that the higher the PB the lower the digit span scores (see also Table 1 for details). We also detected a significant positive correlation between PB and the time required to complete the *switching of attention* task, for both the digits (Swat_D, *p* = 0.018) and the digits + letters (Swat_DL, *p* = 0.031) sub-tests. Therefore, the higher the time required to complete the switching of attention tasks the higher PB. Other significant results include correlations between the EBV score and time required to complete the *switching of attention* task, for the digits (Swat_D, *p* = 0.007) and digits + letters (Swat_DL, *p* = 0.007) sub-tests, and the reaction time variability in the sustained attention task (SaRtSd, *p* = 0.038). No further significant results are reported, although some results approached statistical significance (see Table 2 for details).

**Table 2:** The table reports detailed correlational results between cognitive measures provided in our study and the overall timing performance, measured via PB and EBV.

**Measures**	**PB PTSD**		**PB Controls**		**EBV PTSD**		**EBV Controls**	
Education	*r* = −0.034	*p* = 0.801	*r* = 0.052	*p* *=* 0.688	*r* = 0.030	*p* = 0.824	*r* *=* 0.161	*p* = 0.210
Digitot	*r* = −0.324	*p* = 0.012*	*r* = 0.052	*p* = 0.686	*r* = −0.249	*p* = 0.056	*r* = −0.362	*p* = 0.003*
Digitsp	*r* *=* −0.286	*p* = 0.028*	*r* = −0.009	*p* = 0.941	*r* = −0.176	*p* = 0.182	*r* = −0.447	*p* < 0.001*
Digitot	*r* = −0.298	*p* = 0.022*	*r* = 0.118	*p* = 0.362	*r* = −0.139	*p* = 0.295	*r* = −0.049	*p* = 0.707
Digitsp	*r* = −0.287	*p* = 0.028*	*r* = 0.077	*p* = 0.554	*r* = −0.115	*p* = 0.389	*r* = −0.034	*p* = 0.791
Swat_D	*r* = 0.305	*p* = 0.018*	*r* = −0.133	*p* = 0.301	*r* = 0.344	*p* = 0.007*	*r* = 0.067	*p* = 0.600
Swae_D	*r* *=* 0.079	*p* = 0.547	*r* *=* −0.180	*p* = 0.161	*r* = 0.018	*p* *=* 0.887	*r* = 0.074	*p* = 0.566
Swat_DL	*r* *=* 0.283	*p* = 0.031*	*r* = −0.105	*p* = 0.420	*r* = 0.350	*p* = 0.007*	*r* = 0.145	*p* *=* 0.262
Swae_DL	*r* = 0.131	*p* = 0.323	*r* = 0.096	*p* = 0.458	*r* = 0.129	*p* = 0.333	*r* *=* 0.218	*p* = 0.091
SaRt	*r* = −0.073	*p* = 0.580	*r* = −0.026	*p* *=* 0.839	*r* *=* 0.131	*p* *=* 0.319	*r* = −0.129	*p* = 0.315
SaRtSd	*r* = 0.035	*p* *=* 0.788	*r* < 0.001	*p* = 0.998	*r* = 0.270	*p* = 0.038*	*r* *=* −0.065	*p* = 0.614
SaFa	*r* = 0.073	*p* = 0.580	*r* = 0.107	*p* = 0.405	*r* *=* 0.030	*p* = 0.824	*r* *=* 0.095	*p* = 0.459
SaFm	*r* = 0.081	*p* *=* 0.540	*r* = 0.220	*p* = 0.085	r = 0.136	*p* = 0.406	*r* = −0.032	*p* = 0.804
Satot	*r* = 0.083	*p* *=* 0.530	*r* = 0.242	*p* = 0.057	*r* = 0.194	*p* = 0.236	*r* = 0.023	*p* = 0.858

Please refer to the legend associated to the Table 1 for the details about the acronyms meaning.

#### Controls

We only found two negative correlations with the Digitot: *p* = 0.003 - and the Digitsp: *p* < 0.001 – subtests and the EBV scores. No further significant results are reported (see Table 2 for details,).

## Discussion

This is the first study to our knowledge that offers a systematic examination of time estimation skills in individuals with PTSD. This research aimed to extend previous investigations which reported timing overestimation associated with experiencing highly stressful or negatively arousing stimuli or situations^7,10^. On the basis of this previous research, we predicted that individuals with PTSD would demonstrate an overestimation of time relative to controls. Secondly, we examined the relationship between time estimation capacity and cognitive functioning in PTSD. Our analysis included a range of cognitive measures to specifically evaluate the association of WM and attentional switching with time estimation.

In accordance with our hypothesis, the key finding of our research was evidence for overall time overestimation in PTSD compared to the control participants. From a psychophysics perspective, the current overestimation pattern can be interpreted as a violation of the well documented Vierordts’ law^48^ that predicts a tendency, in healthy humans, to underestimate “long” temporal intervals. In fact, while the timing performance of the control group is characterized by the expected underestimation trend, which linearly increases with the increasing of the temporal interval to be estimated (see Fig. 1), the timing performance of PTSD participants appears only marginally in line with the underestimation pattern predicted by the Vierordts’ law. The time overestimation pattern observed in PTSD is concordant with the evidence of time overestimation is response to negative, highly arousing stimuli compared to positive arousing stimuli^7^, and when experiencing highly arousing and stressful events^10,49,50^. This is in line with evidence of physiological hyperarousal which characterizes PTSD^51^. However, this hypothesis remains speculative, as we did not collect this measure in our participants. It is also interesting to note that the time overestimation pattern reported in our research for the PTSD sample appears similar to data in schizophrenic patients (ref.^52^, for a review^53^). This parallelism is not surprising as the links between childhood trauma, PTSD and psychotic disorders is increasingly recognized^54^, and because in a discrete number of patients these two conditions might even overlap (see ref.^55^). Nevertheless, we are not able to clarify the eventual role played by psychotic symptoms in the time estimation performance of our PTSD sample, as this variable was not measured in the current research. Finally, our results are in line with previous reports^11,12^ documenting a slowed passage of time on individuals with high levels of anxiety.

It is notable that the individuals with PTSD displayed higher intra-individual variability and overall higher inter-individual variability (as sample) in time estimation. The research on inter-hemispheric balance in PTSD might help to explain the higher intra-individual variability for this clinical sample. Electroencephalographic (e.g., ref.^56^), neuroimaging^57,58^ and Transcranial Magnetic Stimulation^59^ studies have shown a right-sided functional prevalence in PTSD. In consequence of this, one could explain the higher timing variability of PTSD as the effect of the hypoactivation of their left hemisphere. This is in agreement with the evidence^60^ of increased timing variability for supra-second durations after cathodal transcranial direct current stimulation, which is known to have inhibitory effects at the cortical level^61–63^, over the left hemisphere. On the other hand, the higher inter-individual variability might be due to other variables related to the clinical condition of the participants (such as extent of hyperarousal symptoms, and extent of dissociative symptoms), which were not explored in the current research.

In line with many previous neuropsychological studies, individuals with PTSD showed evidence of cognitive impairments in both WM, information processing speed (signified by Trials Making A test), and attentional switching (signified by Trails Making B). This outcome replicates previous studies revealing deficits in varying attentional and executive functions, including WM, inhibitory control, attention and cognitive flexibility^64^, attention allocation^65^, information processing speed^66^ and verbal learning and memory^67^, with the largest effects found in verbal immediate memory and attention/WM in a recent meta-analysis^13^. Interestingly, the correlations between cognitive measures and time estimation reported in our study suggest that the time estimation pattern of PTSD, measured via PB, might be linked to the lower WM capacity of these patients (see Table 1 for details). By contrast, the attention performance seems to play a marginal role, as the only significant correlation was detected with the time required to complete the switching of attention task. The timing variability performance of PTSD, measured via PBV, appears predicted by the attention capacity, with particular regard to the time required to complete the switching of attention task (e.g., for both sub-tests) and the reaction time variability in performing the sustained attention task. The negative correlation between the *digitot* measure and PBV in our control participants, which was marginally significant (i.e. p = 0.056) also in the PTSD sample, suggests that cognitive mechanisms underlying this function might play a general role in time estimation variability. Finally, the negative correlation between the *digitsp* and the PBV only for the control group might be interpreted as evidence of a contribution of the recall span functions in time keeping variability, in the context of intact WM skills, as in the case of our control participants.

### Limitations

A limitation in the current study is the absence of measures for the sub-second durations domain. Further limitations are the absence of measures on the patients’ arousal (e.g., skin conductance) and the absence of data on dissociative symptoms and other clinical measures such as depression, anxiety and psychosis, which might help in explaining the origin of the reported time estimation pattern. Future research should examine time estimation in PTSD using experimental tasks with concurrent psychophysiological arousal measures and clinical assessment of arousal and dissociative reactions. Finally, the performance score of 2 participants (1 PTSD and 1 control) was missing with regard to some of the subtests for measuring WM and attention performance.

## Conclusions

To the best of our knowledge, our study represents the first systematic psychophysics investigation of time keeping skills in PTSD. Our results indicate that PTSD is associated with time overestimation, in agreement with clinical reports of slowed down temporal perception during traumatic experiences^68^, and research revealing time overestimation during stressful experiences and negative arousing tasks^7,10^. WM impairments in PTSD were associated with time overestimation, and attentional impairments with the higher variability in estimating the presented temporal intervals. Therefore, it is possible that the temporal dysregulation in PTSD may underlie some cognitive deficits in these two domains, in line with the evidence (e.g., refs^69,70^) of a linking between these variables. Nevertheless, the evidence of time overestimation in PTSD expands our current knowledge about the cognitive alterations of this disorder, and suggests the relevance of this factor as a cognitive marker in assessing the PTSD profile.
